# The Story of the Insane from Year to Year

**Published:** 1903-02-14

**Authors:** 


### THE STORY OF THE INSANE FROM YEAR TO YEAR.

FIFTEENTH ANNUAL SERIES.

### LONDON COUNTY ASYLUM AT CANE HILL, SURREY.

A REDUCTION in the numbers from 2,225 to 2,170 has to be chronicled at this asylum during the past year. There were 329 first admissions, 45 readmissions, 124 discharged recovered, 81 discharged relieved, and 150 died. Calculated on the admissions the recoveries stand at 34·73 per cent. after excluding transfers from other asylums, and calculated on the average number resident the deaths stand at 6·88 per cent. It may be said that both of these percentages are lower than the averages of other county and borough asylums, which stand respectively at 37·14 and 9·94. Of the year's deaths 70 were caused by cerebral and spinal diseases, 20 by consumption, 18 by other lung diseases, and 2 by colitis. Of the year's admissions, the insanity was traceable to moral causes—domestic trouble, mental anxiety, religion, etc.—in 116 instances. Among the physical causes the most potent were intemperance in drink, hereditary influence, epilepsy and previous attacks, accounting respectively for 75, 55, 83, and 93. Large as the asylum is, it is not large enough for the numbers confined in it, and accommodation for 56 patients has been found in the Birmingham asylum. There would seem to have been several cases of colitis during the year, and two patients died of it; but Dr. Moody does not refer to it in his report. We regret to learn that a serious assault was made on Dr. Moody by one of the patients in September 1901. As Dr. Moody was on duty again at the end of the year we presume he has recovered from the effects of the assault, and congratulate him on his recovery. During the year the senior assistant medical officer was promoted to the medical superintendency of the new Manor asylum. The committee record their "appreciation of the efficient manner in which Dr. Moody, his professional colleagues, and the staff generally, have performed their respective duties." The average weekly cost for maintenance was nearly 11s. 6d., being 1s. a week higher than the average of county and borough asylums.

### EARLSWOOD ASYLUM FOR IDIOTS.

ON January 1st this asylum contained 513 patients, and exactly the same number was in residence at the end of the year. There were 47 first admissions, 7 readmissions, 39 discharges, and 15 deaths. Of course the statistics of recoveries and deaths as given in County Asylum Reports, would, in an institution like this, be useless for purposes of comparison with these county asylums, but we may state that the percentage of deaths is very low, only 2·93 on the average number resident. The asylum is, as pointed out by Dr. Caldecott, not only a place where training the higher grades of idiocy can be carried out, but it is a home where all types may find a refuge. Of the 54 cases admitted during the year, 18 were hereditary, 2 were the offspring of consanguineous marriages, and in 20 there was a history of shock or fright to the mother during pregnancy. We should like that Dr. Caldecott could find it practicable to thoroughly investigate these 20 cases, and give us the results of his investigations. We believe that while some might be substantiated, the majority would break down under close examination. When speaking of epilepsy, Dr. Caldecott states that his experience has been that most imbeciles who suffer from epilepsy enjoy better health, and are less liable to attacks of the *status epilepticus* when not subjected to medicinal treatment, and our own experience would lead us to believe that the bromide treatment, which is that referred to by Dr. Caldecott, is very apt to make a quiet epileptic lunatic into a troublesome one, but it also sometimes makes a violent epileptic into a quieter one. The asylum is in one sense a charitable institution; that is, patients are admitted without payment on election by the subscribers, and others by election and part payment. In another sense the Institution is like a private asylum, for patients are admitted by payment alone. We notice that the charitable income was £1,200 in excess of the previous year, and we trust that this will enable the committee to admit paying patients at lower sums, or that the rates of payment will be reduced to some of those already in the asylum who find a difficulty in making the payments. There must be many such.

### LANCASHIRE COUNTY ASYLUM AT RAINHILL.

ON January 1st the Rainhill Asylum contained 2,092 patients, and at the end of the year 11 more were in residence. There were 377 first admissions, 31 readmissions, 135 discharged recovered, 25 discharged relieved, and 214 died. The average number resident during the year was 2,101, and the total number under treatment was 2,495. Calculated on the admissions the recoveries are 33·08 per cent., and calculated on the average number resident the deaths are 10·18 per cent. Of the year's deaths 65 were caused by cerebral and spinal diseases, general paralysis alone accounting for 46 of these. Consumption was the cause of death in 51 cases, other lung diseases in 33, and colitis in 5. Table X. informs us that 66 of the year's admissions owed their insanity to such moral causes as domestic trouble, mental anxiety, religion, and fright. Among the physical causes intemperance in drink accounts for 201, and hereditary influence for 68. Adding these two causes together the sorrowful fact is disclosed that considerably over half of the cases sent to Rainhill during 1901 were from preventible causes, and had no right to exist at all. When will our legislators, our commissioners, our asylum medical officers recognise the fact that the insane percentage of our population is to be perceptibly lowered only by prevention! After showing that over 70 per cent. of the male admissions, and over 26 per cent. of the female were owing to drink, Dr. Wiglesworth speaks out in the following sentences:—"These figures are sufficiently appalling, and give some idea of the enormous amount of preventible insanity that exists amongst us. Everywhere the cry is for increased asylum accommodation. Now asylums are springing up all over the country, old ones are being continually enlarged, and yet all the immense expenditure which this involves might be very largely avoided could men only be got to realise the toxic character of alcohol, and regulate their habits accordingly." To the general public we say, hear, and heed. The average cost per head per week was 9s. 2½d.

### LONDON COUNTY ASYLUM AT HANWELL.

On January 1st 2,532 patients were in residence, and by the end of the year the number had risen to 2,545. There were 396 first admissions, 84 readmissions, 163 discharged recovered, 17 discharged relieved, and 212 deaths. The average number resident was 2,537, and the total number under treatment during the year was 3,012. Calculated on the admissions the recoveries stand at 37·56 per cent., and calculated on the average number resident the deaths were 8·36 per cent. Of the year's deaths 109 were caused by cerebral and spinal diseases, 16 by consumption, 15 by other lung diseases, and 7 by dysentery. Of the year's admissions such moral causes as adverse circumstances, domestic troubles, religion, and mental anxiety were potent in 158 instances, and as is usual among the physical causes intemperance in drink heads the list with 123. Hereditary influence follows with 104, and previous attacks with 104. Dr. Alexander prints a most instructive tabular statement showing the percentages of the admissions for 1898, 1899, 1900, and 1901, in which the two great factors of insanity, drink and heredity, are responsible for these admissions, and it is seen that while the drink percentage among the men in 1898 was only 15, and among the women 8, it has risen in 1901 to 32 per cent. among the men and to 16 per cent. among the women. Heredity has jumped from 26 per cent. in 1898 (taking both sexes) to 48 per cent. in 1901. Such facts are eloquent enough, and although there may possibly be a drop in 1902 from the high figures of 1901, there can be no escape from the unwelcome conclusion that among the lower classes the drink demon, so far from losing power, is increasing it. As Dr. Alexander puts it: "It will be seen from this table what an increasing share alcohol has, directly or indirectly, in the production of insanity, especially in the case of men, and there is no doubt that if we were able to trace satisfactorily the antecedents of every admission, and were not hampered by the wilful suppression on the part of relatives of a drink history, or the non-appreciation by them of what constitutes excess, *the causal influence of alcohol would bulk in our returns even much more largely in our returns.*" The italics are ours, and we gladly print the whole sentence, as it adds force to views that have often been given prominence in these notices. As already stated, there were 7 deaths from dysentery, and it would seem there were in all 64 cases of the disease, 6 being members of the staff. Dr. Alexander has hunted up the old registers of Hanwell, and he finds that during the 64 years ending in 1894 dysentery was a comparatively rare affection, and that during that long period less than half as many deaths occurred from it as in the 7 years ending in 1901. It is curious, too, to note that in the earlier years this form of dysentery was not thought to be infectious; hence cases were not isolated, and yet it did not spread. Many things have been suspected of causing or propagating the disease, but it would hardly be going too far to say that not one of these has been proved. Overcrowding has been often blamed, "but it so happens that from 1895 onwards scarcely a single case occurred in a ward that was overcrowded;" and Dr. Alexander is "disposed to believe that our dysentery of recent years is of a different variety from the sporadic dysentery that occasionally figured in our sick and death returns prior to 1895." In this view probably most asylum medical officers who have had experience of the strangely intractable colitis of the present day will agree. The committee say that "to Dr. Alexander and the medical and general staff our thanks are largely due for the satisfactory condition of the asylum." The weekly cost of maintenance was nearly 10s. 11d. per head.

